# *Our Voice* in a rural community: empowering Colombian adolescents to advocate for school community well-being through citizen science

**DOI:** 10.1186/s12889-022-14559-x

**Published:** 2022-12-22

**Authors:** Felipe Montes, Ana María Guerra, Diana Higuera-Mendieta, Eduardo De La Vega-Taboada, Abby C. King, Ann Banchoff, Augusto César Rodríguez Maturana, Olga L. Sarmiento

**Affiliations:** 1 Department of Industrial Engineering, Universidad de Los Andes, Bogotá, Colombia; 2 Social and Health Complexity Center, Department of Industrial Engineering, Universidad de Los Andes, Bogotá, Colombia; 3 School of Medicine, Universidad de los Andes, Bogotá, Colombia; 4 Department of Psychology, Florida International University, Miami, FL USA; 5 Department of Epidemiology & Population Health, Stanford University School of Medicine, Stanford, CA USA; 6 Department of Medicine (Stanford Prevention Research Center), Stanford University School of Medicine, Stanford, CA USA; 7 Institución Educativa de Santa Ana, Santa Ana, Bolívar, Colombia

**Keywords:** Citizen science, Rural, Well-being, Healthy lifestyles, Built environment, Participatory research, Community, Under-resourced, Latin America, Low-income

## Abstract

**Background:**

Santa Ana is home to an Afro-descendant rural population of the island of Barú in Cartagena, Colombia. While a popular area for tourism, Santa Ana’s population is affected by multidimensional poverty, precarious work conditions, homelessness, broken streets and sewer systems, limited quality education, and a lack of recreation and sport spaces. While Santa Ana’s Community Action Board aims to unify efforts and resources to solve these problems, the state’s capacity to meet the requirements of the Board is limited.

**Methods:**

We evaluated the relationship between healthy lifestyles and characteristics of Santa Ana’s school using the *Our Voice* Citizen Science Research Method. This systemic approach combines information and communication technologies with group facilitation to empower adolescents to: 1) collect and discuss data about factors in their local environments that facilitate or hinder well-being within their school community; 2) identify relevant local stakeholders who could help to address the issues identified; and 3) advocate collectively for local improvements to support increased well-being at a community level.

**Results:**

Eleven citizen scientists ages 13 to 17 years from the science club of Institución Educativa Santa Ana were recruited and together conducted 11 walks within the school to collect data about the facilitators and barriers to student well-being. They identified barriers to well-being related to school infrastructure, furniture, bathrooms, and sense of belonging. They then advocated with school stakeholders and reached agreements on concrete actions to address identified barriers, including fostering a culture among students of caring for school property and presenting their findings to the community action board. This methodology allowed the community to realize how students can become agents of change and take collective action when motivated by solution-oriented methodologies such as *Our Voice.* Project ripple effects, including greater empowerment and participation in collective actions by students, also were observed.

**Conclusions:**

This study underscores the importance of the school’s built environment in the well-being of students in rural areas. The *Our Voice* method provided the opportunity to inform school-based interventions, and promoted ripple effects that expanded productive dialogue to the community level and generated systemic actions involving actors outside of the school community.

## Background

Currently, there are approximately 1.2 billion adolescents, representing 16% of the world’s population. In the coming year/decades, this number is expected to increase, especially in low and middle-income countries (LMIC) [1]. During adolescence, individuals acquire economic, physical, emotional, cognitive and social resources that will define their future and that of subsequent generations [2]. It stands to reason that empowering adolescents within vulnerable communities in ways that may enhance their health and well-being can provide benefits both to the present and future of the population [2].

Evidence shows that about 1.1 million adolescents die annually [3]. The causes of their deaths are typically preventable and are related to factors that include both the physical and social environment and adolescent behaviors [3]. For adolescents aged 10 to 14 years, these deaths are mainly caused by health risks related to water, hygiene and sanitation [3]. For adolescents aged 15 to 19 years, the leading causes of death are traffic accidents, suicide, and interpersonal violence [3]. In both cases, the school environment is pivotal for maintaining health and well-being [2], as adolescents spend a significant portion of their time in school environments. Schools are safe spaces that provide adolescents with resources to develop their skills, address their mental health problems, and improve their social interactions and civic engagement [4, 5]. Nevertheless, in rural communities, school environments are often deteriorated. This diminishes their capabilities as safe spaces for adolescents. Thus, by engaging in improving school environments, adolescents could participate in increasing those capabilities, their sense of belonging to the school, and benefiting from spending time in a safer space.

Santa Ana is a rural district of around 5000 Afro-descendants located on the Island of Barú, Colombia [6, 7]. Over the years, the island of Barú has become one of the most important tourist centers in Colombia. In 2014 a bridge was built to facilitate the entry to the island, and one of the sectors that benefited the most from the construction of this bridge was tourism. As a result, the tourist trade has brought economic opportunities for the population as a whole, but it also has widened socioeconomic disparities and redefined the community as a semi-rural urban-influenced population [8]. Santa Ana is an example of a population with high levels of multidimensional poverty, different forms of violence (e.g. gender-based violence, sexual violence, familial violence, and street violence), lack of decent housing, precarious working conditions, and education of limited quality [8]. In 2019 the United Nations Population Fund reported that in Latin America young people (10 to 24 years old) usually face obstacles in their transition from childhood to adulthood [9]. At this stage of life, young people are faced with problems such as teen pregnancy, unemployment, and lack of access to higher education [9]. For example, just 59.4% of young people have completed high school, the youth unemployment rate is approximately 20, 25% of deaths in young people are due to homicides and only 56% of young people have connected to the internet in the last 3 months [9]. Santa Ana is an exemplar of this broader phenomenon. By 2019, 95.8% of households in Santa Ana lived in poverty and 54.6% in misery [10]. In terms of education, in 2019 the public school *Institución Educativa de Santa Ana* reported a dropout rate of 9.6% (above the average 3.68% dropout rate of Cartagena) and was ranked in the lowest category of academic performance among schools in Cartagena [10]. In terms of health, Santa Ana does not have a primary care hospital, and the hospitals on the island of Barú do not operate properly and require priority intervention [10]. Also, Santa Ana does not have a sewage system, which makes it difficult to manage wastewater, and it has only had aqueduct service since 2017 [10]. Regarding housing, 26% of houses are built with precarious or unstable materials, 51% of the houses do not have walls or the walls are made of fabrics, plastic, cardboard or other waste material, and 49% of the houses do not have a paved floor [10]. Officially, one case of teen pregnancy was reported in Santa Ana for 2018 and 19 cases for 2019. However, this number does not include births performed by midwives and therefore this number may be higher [11]. For the district of Cartagena, in 2021 a problem of adolescent pregnancy was reported since there was a high fertility rate in women between 15 and 19 years old; approximately 63 births occur annually for every 1000 women of reproductive age [12]. Prior research in Santa Ana found that adolescents face situations of violence and substance use that are related to both the physical and social environments that surround them (Guerra AM, et al.: Individual attributes and social network predictors of alcohol consumption susceptibility among adolescents in a rural area of Colombia, unpublished), [13], (Rodriguez AL, et al.: Adolescent violence in rural Colombia: a mixed methods social network analysis, unpublished). These situations are not quantified for Santa Ana since, for example, the official annual report of cases of violence in 2018 is lower than what is expected according to qualitative information from other studies: 2 deaths due to theft, 2 cases of sexual abuse, 3 cases of domestic violence, and 13 cases of interpersonal violence [10].

Given this situation, there is a clear need to improve the well-being of the Santa Ana’s adolescent population in ways that are contextually relevant and sustainable. There are no studies that make it possible to establish the number of adolescents currently in Santa Ana. However, in 2008 a survey was conducted of more than 90% of the population of Santa Ana, which allowed for a determination that the adolescent population represents 14% of the total population of Santa Ana [14]. Currently, Santa Ana is an isolated community that receives little attention from state agencies. There is a noticeable lack of coordination between the entities at the regional and local levels [6], making it difficult to develop and implement stable policies and planning at the local level [6]. As a result, many citizens have lost interest in becoming involved in local processes and dynamics that could positively impact the well-being of the community [6]. Such a circumstance has been conceptualized as being compromised by limited “social capital,” wherein community stakeholders have lost the capacity for cooperation, trust, and coordination [15].

In the above context, citizen science “by the people,” as exemplified by the *Our Voice* citizen science research method, allows residents the unique opportunity of participating directly in collecting meaningful information about their local contexts, collectively analyzing and prioritizing their information, and then developing and collaborating to enact relevant actions based on their results [16].

The use of mobile technology among adolescents is increasing. Currently, approximately 80% of adolescents in the United States have a mobile phone and use it frequently [17]. In Colombia, it is estimated that 5 out of 10 adolescents have a mobile phone [18]. Previous studies have shown that the use of mobile technology may provide a way to engage adolescents in projects where they express their perspectives and advocate for issues that affect them [19–23]. *Our Voice* uses mobile technology as a way of engaging adolescents and other groups in the process of identifying local barriers to and facilitators of health and well-being. The web-based *Our Voice* data platform then allows for the creation of community reports that enable citizen scientists to draw on their own data in collaborating to promote changes in their local environments.

One of the main objectives of *Our Voice* is to provide populations that traditionally have not had the opportunity to be heard with the means for collectively participating in community decision-making [16]. This model has been applied in over 20 countries spanning 6 continents and has been utilized by different age groups in varying contexts and circumstances to address local problems that influence the well-being of the community [16, 24]. However, it has never been applied in a rural area with a Latin American population–particularly one of African descent. This article documents the application of *Our Voice* in Santa Ana, Barú. The purpose of this study was to evaluate the impacts of this type of community-engaged citizen science method among the adolescents of Santa Ana, particularly with respect to the school environment, and to explore how students might use their group data to advocate for change with school stakeholders. The results were aimed at informing decision-makers about possible interventions for improving the well-being of the adolescent population of Santa Ana as well as similar populations.

## Methods

### Study setting and population

The island of Barú is composed of three communities: Barú, Ararca, and Santa Ana. Santa Ana’s population is approximately 5000 inhabitants [7]. More than 80% of the population self-identifies as Afro-descendant [8]. As noted earlier, while Santa Ana is administratively dependent on the city of Cartagena, this district is geographically isolated and therefore the capacity for intervention by the state has been limited [6].

### Study design

This is a descriptive study and is a first-generation formative pilot study to evaluate the initial feasibility and utility of using this type of technology-enabled “by the people” citizen science method among this underserved and understudied population.

**Fig. 1:**
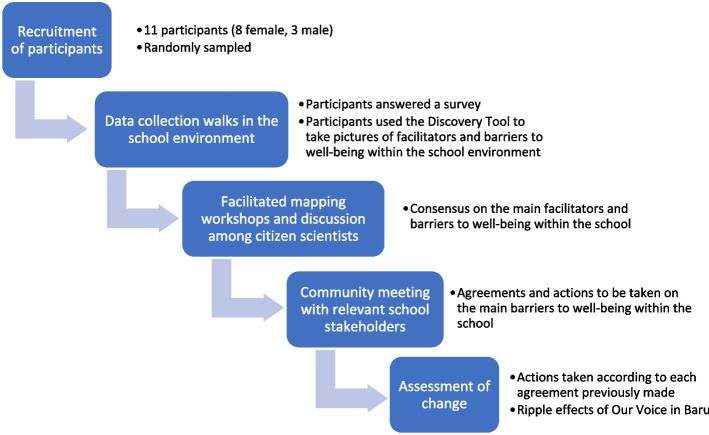
Our Voice method in Barú

This study was developed using the *Our Voice* method developed at Stanford University (Fig. 1), which consists of the following phases: 1) recruitment of participants; 2) technology-enabled data collection during community walks within the school property; 3) facilitated participatory mapping workshops and discussion among citizen scientists to build consensus around those issues of highest priority that could be feasible to address; 4) a participatory community meeting with relevant school stakeholders; and 5) assessment of change.

### Sample – recruitment of participants

In this first-generation study, a total of 11 participants (8 female and 3 male) were randomly selected in a raffle among students of the science club that is composed of students of different high school levels. Previous *Our Voice* studies reported that this number is typically sufficient to attain group consensus on the major barriers and enablers in a specific locale [25]. All the selected adolescents and their parents or caregivers gave their written consent to participate in the study. This study was reviewed and approved by the Institutional Review Board of the Universidad de Los Andes in Bogotá (Minutes 945 of 2018).

### Data collection

#### Stanford Discovery Tool

The Stanford Discovery Tool is a mobile application-based environment assessment tool that residents use to collect information about features of their local environments that impact health and well-being [26]. Deployed successfully by individuals ages 9 to over 95 years old across different countries, including Colombia [16, 25], this tool allows citizen scientists to record geotagged photos, comments, and ratings that are then uploaded to a secure server where collective data reports can be generated. As part of the Discovery Tool app, participants completed a brief survey that included a standard question rating their overall health relative to others of a similar age and sex on a 5-point scale from poor to excellent [27], and several frequently used questions about perceptions of cohesion and empowerment in the community using Likert scales from the standard *Our Voice* protocol [27]. The anonymous, de-identified Discovery Tool data collected during the walks were stored on a secure server at Stanford University, accessible to study investigators via a password-protected web interface.

#### Walks within the school

The data collection walks were carried out across 3 days. Before data collection, the citizen scientists were provided with a cell phone that had the Discovery Tool installed, and given verbal instructions on how to use it. Trained research staff made sure that each citizen scientist knew how to use the phone and Discovery Tool app, and remained in proximity to the citizen scientists on the walks in case they had technical difficulties, while avoiding any interference with data collection. Following completion of the participation agreement and informed consent form, each citizen scientist opened the application, read a welcome message and safety suggestions, with support of the research team if required, and took an individual walk in their school site with the goal of answering the following question: “What things facilitate or hinder your well-being and that of your community at the Santa Ana Educational Institution?” The research staff designed the question with the Amor Por Barú Foundation staff and validated its comprehension with several adolescents before starting the fieldwork. The walks took place inside the school and each citizen scientist defined their own route, decided whether they want to include interior and/or exterior components of the school facilities, and determined when the walk would end. Throughout the walk, the citizen scientists took photos of the elements of their built environment of relevance to this research question. For each element, they selected in the app whether it was considered a facilitator of their well-being (happy face emoticon), a barrier to their well-being (sad face emoticon), or both. They then used the app’s audio-recording feature and/or text comments to describe why they took the picture. At the end of the walk, each participant completed a short survey in the Discovery Tool consisting of 8 questions regarding demographic data, perceptions of health, perception of the level of support among school site members, levels of individual and group empowerment in decisions that affect the community, and level of knowledge about relevant actors/stakeholders (e.g., important hotels in the area, the community board) in the larger community.

### Facilitated mapping workshops and solution-building among citizen scientists

#### School mommunity meeting 1: participatory mapping workshop and building consensus on perceived facilitators and barriers to well-being

Once the walks were finished, study investigators processed the Discovery Tool data to create collective reports for participant review. At a facilitated meeting, each participant was given a folder containing printed copies of their geotagged photographs accompanied by verbatim transcripts of their text and/or audio comments as well as map locations and positive or negative ratings. The discussion among citizen scientists was facilitated by the research team and in the context of a school mapmaking activity in which adolescents constructed their own map of the school.

During this community mapping activity, participants discussed their findings, found common ground, and set priorities to improve well-being in their school environment. To facilitate this process, citizen scientists were divided into two groups and jointly drew school maps where they identified natural, political, and social elements related to well-being. Afterward, they were asked to add another layer to the map, consisting of the photos taken with the Discovery Tool. Then, the two groups presented the maps to each other and worked together to identify and prioritize the main barriers and facilitators of well-being in the school. Near the end of the workshop, the citizen scientists proposed possible solutions for each of the main barriers identified. Using this information, they were then provided with mentorship by the study staff to prepare a presentation using slides, so that they could present their findings at a subsequent meeting with the relevant decision-makers of the school.

This method of community mapping invites citizen scientists to become active participants in sharing their experiences and developing a shared vision of their school, thus facilitating feelings of empowerment [28]. During the workshop, the participants were encouraged to make distinctions related to their built and local environment from different points of view. They were encouraged to be observers within their system when they carried out the walks, and then observers from outside their system when they built their map of the school. This is important because, according to second-order cybernetics, the system’s distinctions let the observer define the meaning of what is observed and analyze it [29, 30].

### Community meeting with relevant school stakeholders

#### School community meeting 2: advocating and reaching agreement about changes in the school community

Following the first community meeting, the research team organized a school community meeting with the relevant stakeholders of the school. The meeting was attended by the directors of the school, the teachers, the architect in charge of future improvements to the school’s infrastructure, the Amor por Barú Foundation Team (i.e., the executive director of this non-governmental organization [NGO]), two coordinators that provided logistic support to the *Our Voice* process, and a landscape contractor), and the Universidad de Los Andes research team. In this meeting, citizen scientists presented their findings and proposals for potential solutions, which were discussed with the stakeholders to reach agreements on relevant actions to take. After this community meeting, the research team prepared a report summarizing and documenting the findings and agreements that resulted from the project.

### Assessment of change

#### Ripple effects and outcomes of *our voice* in Barú

Six months following the second community meeting, the research team developed a report for the school directors describing the results of the *Our Voice* process. The report consisted of a review of the different stages of the process, a listing of the barriers and facilitators identified, the ideas generated by the citizen scientists about how to improve the general well-being of the school community, and the commitments that were agreed upon between decision-makers and citizen scientists at the second community meeting. Also, a descriptive statistical analysis was conducted and presented with the quantitative data that the survey yielded.

The action plan was subsequently tracked and the activities and their results were monitored both by the Amor por Barú Foundation and the school by e-mail. A year later, though school activities were suspended due to the COVID-19 pandemic, researchers conducted a semi-structured virtual interview with the principal of the school and the staff at the Amor por Barú Foundation provided a report via e-mail. The research team inquired about the effects that the project had on the school community and the actions carried out for each of the points that were agreed upon in the community meeting.

### Qualitative data integration and analysis

All information gathered by citizen scientists using the Discovery Tool was transcribed and downloaded with the prior authorization of citizen scientists and their parents/caregivers through informed consent forms. Each of the workshops and community meetings was documented through field notes by the research team that were later transcribed and organized by the research team. The results from the different stages were then structured into a table, and the research team classified them into topics. The information obtained through the virtual semi-structured interview with the principal of the school was recorded and later transcribed. Lastly, to analyze the effects that the project had on the school community, the results of the interview with the principal and the report of Amor por Barú Foundation were compared with the points agreed upon in the community meeting.

## Results

### Recruitment of participants and data collection walks within the school

#### Descriptive characteristics of citizen scientists

**Fig. 2:**
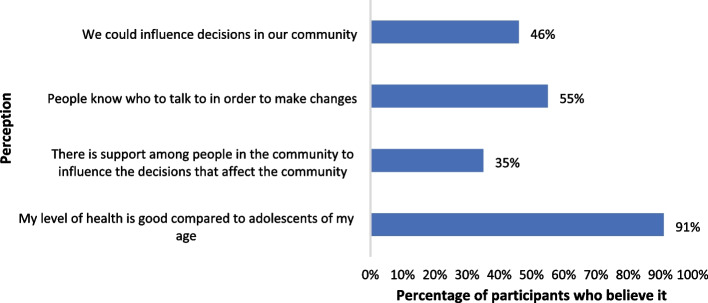
Results of the survey conducted before the walks with the Discovery Tool

Eleven high school citizen scientists between the ages of 13 and 17 participated in the study, 7 of them female. Figure 2 shows the results of the survey conducted before the walks. The majority of the citizen scientists considered their level of health to be good compared to adolescents of the same age, yet there was a low perception of empowerment and community cohesion. At the same time, the participants believed that people in their community know whom to talk to in order to make changes, and that together they could influence decisions in their community (with 35% not believing this and 18% having a neutral position on it) (Fig. 2).

### Facilitated mapping workshops and solution-building among citizen scientists

#### Community meeting 1: participatory mapping workshop and building consensus on perceived facilitators and barriers to community well-being

Citizen scientists met at the facilities of the Fundación Amor por Barú. Ten of the 11 citizen scientists who made the walks inside the school attended the meeting (7 girls and 3 boys). In the half-day meeting, the citizen scientists discussed the concept of school and neighborhood environment territory and well-being in order to understand the topics that would be discussed in the workshop; identified the barriers and facilitators to achieving well-being in the school environment; reached consensus on the actors/stakeholders involved in potential solution-building; and represented on a map the relationships between the actors and the places where facilitators and barriers were observed.

**Fig. 3:**
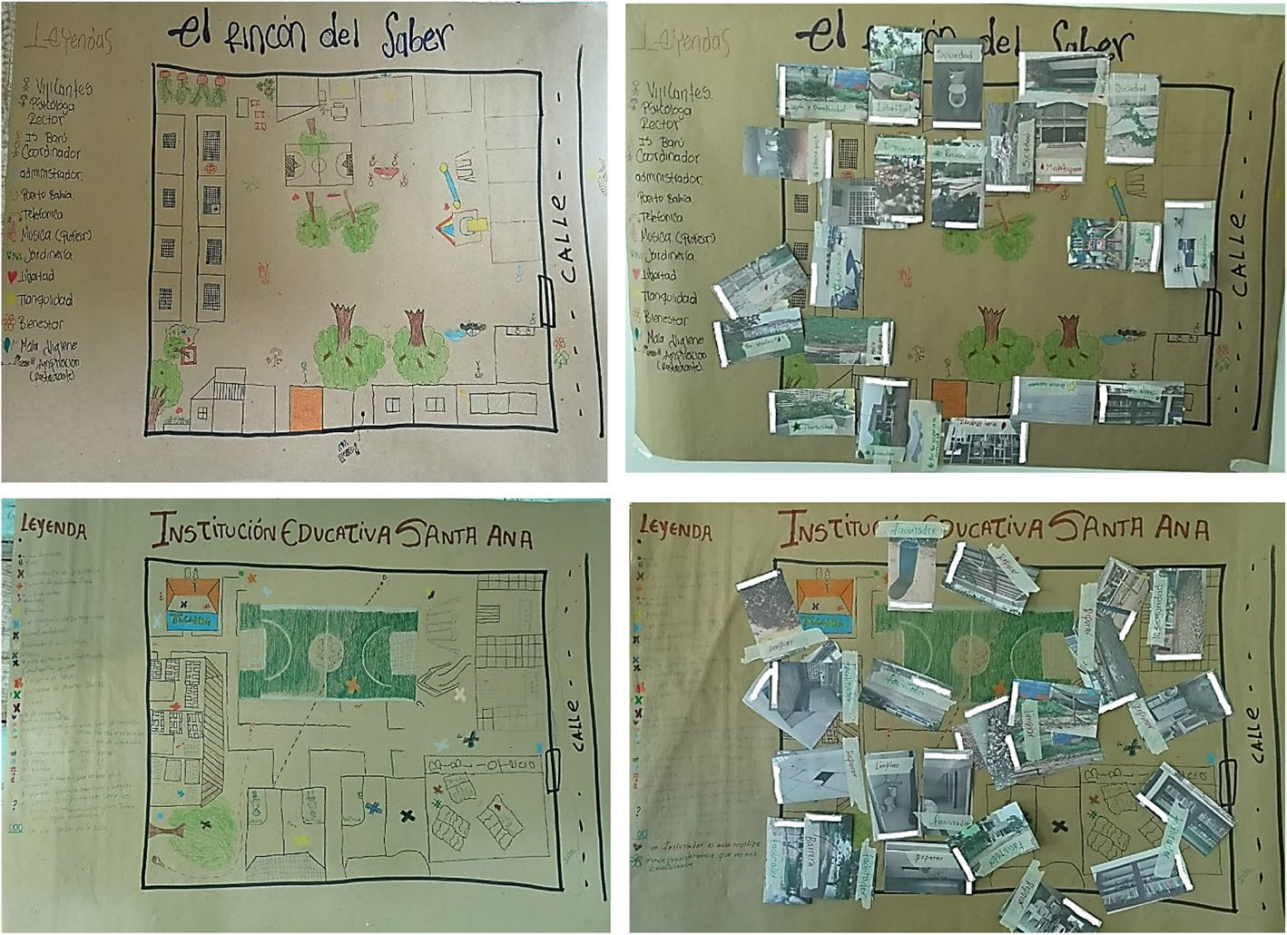
Mapmaking activity. On the left are the maps drawn by the participants and on the right the photos taken during the walks in relation to the elements and places highlighted on the map

Participants identified the following facilitators of achieving well-being in the school environment: the library; the classrooms dedicated to vocational education and training (VET); the nearby park and green areas on the school grounds; bathrooms that were clean and in good condition; the orchard on the school property; available trash cans as factors that help to keep the school clean; the soccer field, which facilitated physical activity; and the school store where students can buy food. In addition, participants identified the following barriers to achieving well-being in the school environment: dirty common zones; poor condition of the recreation area a low-rise wall around the school property, which can lead to falls and injury; insufficient or poorly maintained urinals and bathrooms; poor condition of the indoor fans; poor condition of the eating area chairs and tables; and danger and risk of personal injury from debris, broken walls, trash, damaged fans, and damaged chairs and tables. All of the facilitators and barriers mentioned above were placed on the two maps utilizing the photos taken during the walks (Fig. 3).

Next, citizen scientists prioritized the main barriers and facilitators to achieving well-being in the school environment. They reported that the main barriers to their well-being in the school environment were related to danger or the risk of personal injury; insufficient and poorly maintained urinals and bathrooms; the poor condition of chairs and tables; and the risk taken by students climbing over the low walls of the school. Citizen scientists stated that they perceived additional personal risks related to the presence of debris and damage to the school’s physical infrastructure. One citizen scientist expressed: *“For me, the blocks [referring to debris located in the recreation area] that are there can cause damage. For example… they can injure a foot or they can fall and hit your head.”* When citizen scientists referred to insufficient and poorly maintained urinals, they stated that only a few of the urinals were in good condition and could be used while the others were either damaged or dirty. Also, citizen scientists stated that at the back of the school there is a low wall that presents a risk for students because some try to leave school by climbing over it and can fall and hurt themselves. One citizen scientist explained: *“I don’t like this side of the school grounds because there is a wall and many children leave at rest time and that affects us because they can climb the wall and get hurt and that’s why I don’t like it”.*

To address the barriers prioritized by the citizen scientists, the group proposed solutions and discussed ways that citizen scientists could contribute to change. These solutions are summarized in Table 1.

**Table 1:** Prioritized barriers and solutions proposed by Citizen Scientists during the Participatory mapping workshop

Barriers	Solutions proposed by Citizen Scientists	Pictures taken by Citizen Scientists
Danger and risk of personal injury from debris, broken walls, trash, damaged fans, damaged chairs and tables	• Clean and organize the school facilities, such as the classrooms, to increase safety • Move debris that could be a tripping hazard, with students helping to organize and move the debris	
Insufficient or poorly maintained urinals	• Replace the boys’ bathroom urinals and repair the problem of stagnant water • The students potentially contributing to this activity with their labor	
Poor condition of the eating area chairs and tables	• Provide feedback and guidance to students who damage chairs and tables during fights	
Low-rise wall, which can lead to falls and injury	• Raise the wall to prevent students from climbing on or over them • Have more guards in the area so that students do not try to leave the school grounds in that manner	

The citizen scientists reported that the main facilitators of well-being in the school environment were the library; the classrooms dedicated to vocational education and training (VET); and the green areas (the garden and the orchard) (see Fig. 3). The library was valued by citizen scientists because they considered it to be a space associated with tranquility and the possibility of learning. One citizen scientist stated: *“I like this part of the school environment because it brings us peace of mind, it teaches us a lot and it is always neat and clean.”* In addition, VET classrooms were perceived as positive because they provided citizen scientists the opportunity to be trained in technical education that might afford them job opportunities at the end of school. One citizen scientist explained it in this way: *“The vocational training is good because we need it so that later we have funds to pay for our studies and livelihood …it helps us a lot in getting ahead”.* Finally, green areas were associated with freedom, recreation, and tranquility. Trees were considered to be a good meeting place to avoid the sun during recess hours, and gardens were valued because, as one student described, they “give life to the school.”

### Community meeting with relevant school stakeholders

#### School community meeting 2: advocating for and reaching agreements about changes in the community

Citizen scientists presented to school stakeholders their findings and possible solutions for the main barriers to well-being that they had identified during the previous meeting. For each of the solutions proposed, the causes and solutions were discussed, and action steps were identified and agreed upon by the attendees as a group. During the conversation, a barrier emerged that had not resulted from the walks but that was important for the school community: High traffic speed on the main street near the school. Table 2 shows a summary of each of the topics discussed, together with the relevant agreements and action plans.

**Table 2:** Action plan agreed between citizen scientists and the school decision-makers during School community meeting 2

Barriers	Agreements and actions plan
Diminished sense of belonging that leads to the destruction of school facilities	• Teachers committed to discussing with students the importance of taking care of things in the school environment • The school will provide psychological support to students for anger management to reduce the destruction of school property • The school will develop appropriate signage and messages promoting a greater sense of belonging • The school will explore the creation of a system of fines to be used as a sanction to discourage destructive behavior and promote a sense of belonging
Danger and risk of personal injury on the school grounds due to debris, etc., and insufficient or poorly maintained urinals	• Cleaning days will be organized by the school staff, and students will be encouraged to participate • During cleaning days, school staff will give talks to students to raise awareness about taking care of school grounds
The poor condition of the eating area chairs and tables	• To reduce the destruction of facilities, the school administrators committed to provide psychological support to students for anger management
Low-rise wall	• The school principal stated that the law does not allow increasing the height of the wall • Teachers agreed to comply with the surveillance schedules that have been assigned to them • Students will participate in surveillance activities at appropriate times during the day • The school administration committed to explain the consequences of school non-attendance and dropout to the student body to diminish the frequency of students’ attempts to leave school by climbing over the wall
High traffic speed on the main street near the school	• The school will ask for support from the general community to reduce the speed of vehicles on this street • The school will install caution signs to raise awareness among community members related to respecting speed regulations • Students will draft a letter asking the traffic police for help
There is not support among people in the community to influence the decisions that affect the community: Communication of project findings and future meetings	• The school believes that students should show their findings to the whole school • Citizen scientists also should present their findings to the greater community and the Santa Ana assembly • Citizen scientists need to become leaders to bring change to the entire Santa Ana community • The director of the Amor Por Barú Foundation will take these findings to other community stakeholders

### Assessment of change

#### Effects and outcomes of *our voice* in Barú

**Fig. 4:**
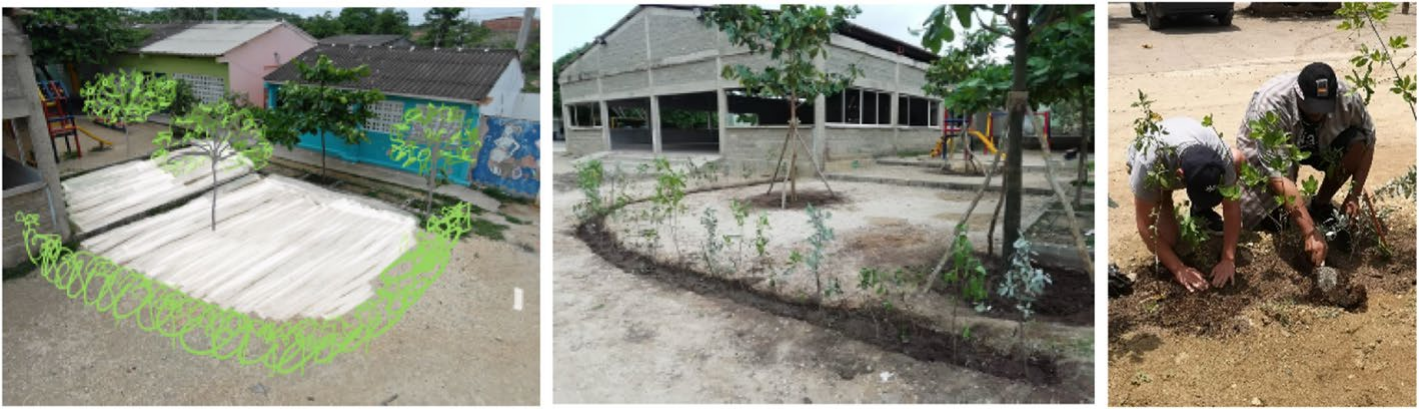
Urban acupuncture executed by the Amor por Barú Foundation

Six months after the second community meeting, the Amor por Barú Foundation had instituted several actions related to reducing school infrastructure danger and risk of personal injury identified by the citizen scientists. Figure 4 shows how the Foundation removed debris and carried out containment (and beautification) of spaces by adding vegetation on the school grounds. The project was named “urban acupuncture.”

Then, 1 year later, the research team followed up on the results and actions taken, conducting a semi-structured interview with the school principal to identify further the types of effects that *Our Voice* in Barú had on the school community. The principal noted that the major effects of using this methodology were to allow the community to realize that students can be empowered and take collective action when they are motivated by participative solution-oriented methodologies such as *Our Voice.* The principal also commented that a “ripple effect” emerged within the teaching staff as they replicated various aspects of this methodology in their classes. For example, in the elementary school, the students made expeditions to the local area near the school to visually identify the characteristics of its flora and fauna. Other major effects of this initial *Our Voice* project in Barú have included the general empowerment of the students beyond those participating initially as citizen scientists. The principal mentioned that the students have been taking more collective action, with a greater number running for the student council and starting to talk to more community groups like the local fishermen’s group and the Santa Ana community board about issues that concern them. Table 3 presents the action steps that have been carried out for each of the topics discussed in the meeting between the school and citizen scientists.

**Table 3:** Verified actions taken as a result of plans agreed upon citizen scientists and school decision-makers

Barriers	Actions taken
Diminished sense of belonging that leads to the destruction of school facilities	• The student council organized care groups at recess and in the afternoon to improve feelings of student belonging
Danger and risk of personal injury and Insufficient or poorly maintained urinals and bathrooms	• The students scheduled cleaning campaigns in the areas that generated problems • An architect worked on solving the stagnant water issue • The parking lot was organized to minimize the risk of personal injury • Restroom damage was fixed and a new adult restroom was built
The poor condition of the eating area chairs and tables	• No action was reported
Low-rise wall	• No action was reported
High traffic speed on the main street near the school	• The police promised to assign a traffic policeman to provide education related to road behavior
There is not support among people in the community to influence the decisions that affect the community: Communication of project findings and future meetings	• The findings were presented to the Youth Social Forum. This is a school event where students present different situations and problems to the entire Santa Ana community
Additional actions/effects	• The students’ group for ecological expeditions was strengthened based on the findings. Students and teachers began to use aspects of the Our Voice methodology to collect information and present the findings of the expeditions • The Our Voice methodology was brought to school courses as a learning methodology • The students spoke with the group of local fishermen about the importance of the garden in the school as a facilitator of student well-being and got the most important hotel in the area to give them plants and fertilized soil for the garden • Now there are more applications to the student council • There are more collective actions and cooperation, as described above • The activity “let’s talk with my principal” was created to increase trust between students and school directors

## Discussion

The *Our Voice* in Barú study is, as far as we are aware, the first citizen science project in a rural Latin American context focused on supporting members of a school community to advocate for data-informed changes in their local environment to increase well-being. During this study, the adolescents identified and presented to decision-makers what they perceived to be the most relevant barriers and facilitators of the built-environment for well-being in their school. The main barriers reported were the poor or insufficient condition of the school infrastructure and the lack of surveillance of risks to students, all of which impacted the low sense of student belonging. In the community meeting, citizen scientists explained that these barriers were related to their well-being, as adolescents are exposed to injuries, fights, and at risk of school dropout. At the same time, there is a reinforcement loop because school facilities are often damaged during fights, and as violent behavior continues to be normalized, school facilities continue to be damaged. As mentioned above, Santa Ana is a place where there is a presence of different forms of violence (e.g. gender-based violence, sexual violence, familial violence, and street violence) [8]. Another reinforcement loop occurs between the lack of risk surveillance for student injuries and school dropout. The problem reported about the school wall through which students escape reveals the concern that students want to leave the school to carry out other activities. According to the latest report on quality of life on the Barú peninsula, the dropout rate at the Institución Educativa Santa Ana was 9.6% in 2018, 2.5 percentage points more than in 2017 [10]. In addition, the average time of school study in Santa Ana is 5.45 years, and is expected to be at least 10 years old to complete the school stage [10]. So, if there are incentives outside the school for adolescents not to want to study, and there is a lack of surveillance of risks to students, they will try to escape more from the school and will be at greater risk of harming themselves in the attempt. Furthermore, as more students drop out, others are implicitly encouraged to do so. Adolescents in Santa Ana usually often stop studying to go to work and provide income for their homes. This may be related to the fact that the average study time in Santa Ana is much shorter than in Cartagena. Thus, the findings of citizen scientists about barriers in the built environment reveal problems related to the well-being of adolescents in Santa Ana that need to be addressed. Interestingly, the solutions and action agreements reached in the community meeting with stakeholders are focused not only on fixing the built environment, but also on dealing with the underlying problems that were mentioned above (i.e. school dropout, risks for students, violence). The citizen scientists also identified facilitators of well-being related to the presence of green areas and spaces supportive of studying. These facilitators are again related to the well-being of adolescents to the extent that they could be considered safe spaces for them. These are physical spaces where adolescents can meet without being exposed to the difficult challenges that generally affect them in their environment, such as violence, substance use, discrimination, and teen pregnancy, among others [8, 10]. In addition, the spaces supportive of studying reveal the willingness of adolescents to study and the need to address the problem of school dropout that is evident in Santa Ana. Through the *Our Voice* process, the citizen scientists had the opportunity to have their perspectives on these issues be directly heard by the decision-makers at their school. This positive dynamic helped to close the communication and support gap for students who reported at the beginning of the project that they felt little to no support among people in the community and did not feel that they could influence the decisions that affect them. As elicited in the follow-up with the school principal, this exercise allowed the students to realize that this is not always true, and that they can empower themselves to advocate for changes by establishing mechanisms of participation. Concerning this, it is useful to note that Santa Ana is a community characterized by having strong, largely hierarchical power relations and having few participatory mechanisms due to the low attention of the state [8]. In this respect, it is useful to highlight the activity of participatory mapping, reported in the social sciences to be an exercise that can transform power relations so that they could be inclusive and community-based [28]. Projects such as this have the potential to shorten distances between decision makers and communities.

Interestingly, comparisons with other *Our Voice* studies conducted in schools in both urban Colombia and the US point to similarities in the facilitators and barriers identified. In a school study carried out in Bogotá, Colombia, for example, green areas were also identified as facilitators for a healthy life and similar barriers emerged such as low maintenance of bathrooms and classrooms and lack of a culture/norms to keep the school environment in good condition [24]. In Barú, one of the barriers that emerged in the conversation between stakeholders and citizen scientists was car traffic on the school’s main road. This finding has been reported in other school-based studies in different countries and contexts that have focused on identifying facilitators and barriers to having safe routes for students to travel to school [27, 31, 32]. For example, in a safe routes to school study in the US, some of the barriers that emerged were also related to traffic and speed on the streets near the school [31]. To address this issue in Barú, an agreement was reached with the police to facilitate targeted driver education programs. To date, however, this has not happened. In the US study, the solutions to improve school traffic were not pursued because the key municipal transportation stakeholder for the area left his/her position [31]. This suggests that it is important that agreements do not depend on specific actors but, rather, should occur at the organizational level and formalized, whenever possible, within each entity. An example of organization-level action occurred in a study occurring in Bogotá, Colombia where the citizen scientists were trained by members of a nearby law school in culturally relevant advocacy skills to help advance their change efforts [24].

Ripple effects identified after the project ended indicate that the *Our Voice* project in Barú has the potential to facilitate changes at a systems level in the broader Santa Ana community, and has generated emergent phenomena such as cohesion, cooperation, and coordination among students, directors, teachers, and external community members. As the principal of the school noted 6–12 months after project completion, following the *Our Voice* intervention students began to more fully and frequently engage in collective action with important groups in the community such as fishermen, the major hotels in the area, and the Santa Ana community board, where the results of the study were presented. Of note, an *Our Voice* study in Bogotá, Colombia aimed at enhancing local parks for physical activity and well-being also showed similar ripple effects [33]. In that study, the citizen scientists’ results were presented to additional groups that were not involved initially, and the *Our Voice* approach helped to promote dialogue among different community actors [33].

The presence and actions of local organizations such as the Amor por Barú Foundation were important factors that promoted these changes at a systems level. An actor like the Foundation has the power to create synergies across different community organizations and entities by acting as a bridge and closing communication gaps. Findings from *Our Voice* in Baru are also consistent with the health-related citizen science literature more generally. A recent review of this literature suggests that this type of citizen science approach can be helpful in addressing both physical and social environmental determinants that affect well-being by empowering people to identify and change the community-level factors that affect their well-being [34].

This study has several limitations. First, while the citizen scientists were randomly selected from the student pool comprising the science club of the school, which can help to reduce bias related to self-selection, the club itself represents a select group of students who may not be representative of the larger school population. Although this could bias the results of the study, a previous study suggested that having a greater number of individuals who are leaders could enable a better advocacy phase as they are agents of change [24]. In addition, the pool of students was composed of students of different sex and age groups, so the selection process helped to preserve student diversity for these categories. Second, we were unable to fully monitor the progress made around the project action plan that was developed, as the school’s activities were suspended due to the pandemic caused by the COVID-19 virus. It was not possible at that point to organize interviews with students and teachers to get their perspectives, as flight suspensions across the country made it impossible to travel. Further, we were unable to organize virtual interviews due to the lack of internet access within the Barú population. However, we were able to speak with the principal, and the Amor por Barú Foundation sent us a report of the subsequent citizen science-inspired activities occurring in the school. Also, we remain in contact with the school and continue to develop different projects in Santa Ana that are aimed at improving the well-being of the population and its built environment. In particular, in 2022 we will finish a Our Voice project with walks outside the school and this allows us to continue monitoring the improvement of the school environment. These methods provided us with valuable information concerning the school’s progress in responding to student concerns and enhancing the school environment in support of well-being. Finally, due to the current political milieu in Santa Ana, we could not bring the findings to a local governmental audience. The political conditions in Santa Ana are complex as its governance is divided and it is a territory where there is still widespread violence. These issues notwithstanding, we received strong support a strong commitment from the school authorities to promote student-identified changes within the school environment.

## Conclusion

This study highlights the impact of the school built and social environments on the well-being of students in a rural area of Colombia. Young people in such environments remain a largely untapped resource for generating both meaningful data concerning contextual barriers to well-being and practical multi-sectoral solutions. *Our Voice* provided the opportunity to actively engage students in informing interventions that could be enacted in the school environment and successfully promoted dialogue between directors, teachers, and students. The project had ripple effects that expanded this dialogue to the broader community level and generated systemic actions that involved actors outside of the school community. Such participatory action forms of citizen science are feasible to conduct in such under-resourced rural communities and worthy of larger-scale investigation.

## Data Availability

The datasets generated and/or analyzed during the current study are not publicly available due to the data management policies established in the research protocol approved by the ethics committee but are available from the corresponding author on reasonable request.

## Abbreviations

LMIC: Low and middle-income countries
NGO: Non-governmental organization
